# Caudate Head Ischemic Stroke with Concurrent Tubercular Meningoencephalitis: A Case Report

**DOI:** 10.3390/reports8020055

**Published:** 2025-04-23

**Authors:** Filippo Nuti, Giulia Ruocco, Patrizia Pasculli, Maria Rosa Ciardi, Giovanni Fabbrini, Matteo Bologna

**Affiliations:** 1 Department of Human Neurosciences, Sapienza University of Rome, 00185 Rome, Italy; 2 Department of Public Health and Infectious Diseases, Sapienza University of Rome, 00185 Rome, Italy; 3 IRCCS Neuromed, 86170 Pozzilli, Italy

**Keywords:** tubercular meningoencephalitis, ischemic stroke, meningoencephalitis complications, stroke secondary prevention

## Abstract

**Background and Clinical Significance**: The pathogenesis of ischemic lesions in tubercular meningoencephalitis remains unclear, as do the best therapeutic strategies during the acute phase and for secondary prevention. **Case Presentation**: We report on an atypical case of tubercular meningoencephalitis with a concomitant ischemic stroke. The infectious origin of the ischemic lesion was hypothesized due to a discrepancy between clinical and radiological findings. The patient underwent neuroimaging, blood tests, and a lumbar puncture to diagnose tubercular meningoencephalitis. She subsequently started on antitubercular therapy. Despite the initiation of treatment, her neurological condition worsened. A computed tomography revealed hydrocephalus, leading to the placement of an external ventricular shunt. This intervention resulted in a reduction in ventricular size and an overall improvement in her clinical condition. To reduce the risk of death, secondary prophylaxis with cardioaspirin was added to her treatment regimen. **Conclusions**: This report highlights the diagnostic and therapeutic challenges encountered in managing patients with tubercular meningitis presenting with concomitant ischemic stroke. By elucidating the complexities of this clinical scenario, we emphasize the importance of early recognition, comprehensive evaluation, and multidisciplinary management to optimize patient outcomes.

## 1. Introduction and Clinical Significance

The presentation of ischemic stroke varies widely depending on the affected cerebral vascular territory and underlying etiology. While ischemic strokes involving the basal ganglia—particularly the head of the caudate nucleus—are relatively rare, they present significant diagnostic and management challenges due to their diverse clinical manifestations [1,2,3].

This case highlights the importance of considering alternative causes of ischemic brain lesions, especially when there is a discrepancy between clinical presentation and neurological examination findings. Notably, if the stroke occurs in the “Tubercular Zone” (TB zone), clinicians should be vigilant for tuberculosis as a potential differential diagnosis [1,4,5,6]. This is particularly relevant in patients presenting with typical neurological symptoms such as headache, altered sensorium, and confusion. Even in non-endemic regions, a patient’s travel history or origin from a high-prevalence area should prompt the consideration of tuberculosis in the diagnostic workup [7,8].

## 2. Case Presentation

### 2.1. Patient Information

Demographics:

A 58 year-old Filipino woman living in Italy for approximately 20 years.

Presenting Concerns:

The patient presented with generalized weakness and a five-day history of headaches unresponsive to non-steroidal anti-inflammatory drugs (NSAIDs).

Medical History:

Hypothyroidism.

Family and Psychosocial History:

The patient had been residing in Italy for two decades. She visited the Philippines two years ago.

Relevant Past Interventions:

No significant prior medical interventions noted.

### 2.2. Clinical Case

We present the case of a 58-year-old Filipino woman who came to the emergency department with generalized weakness and a five-day history of headaches that did not respond to non-steroidal anti-inflammatory drugs. Her past medical history was notable only for hypothyroidism. She had been residing in Italy for approximately 20 years and worked as a housekeeper, with her last visit to the Philippines being two years prior. A chronological summary of the clinical presentation, diagnostic findings, and therapeutic interventions is provided in Table 1. 

**Figure 1:**
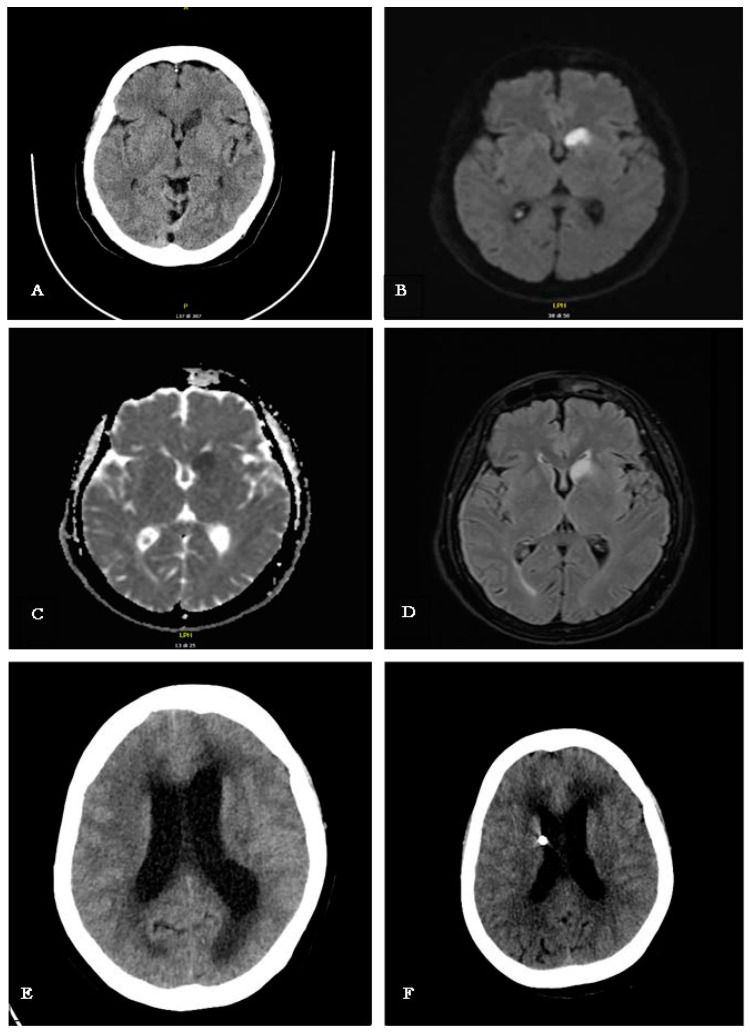
(**A**) CT scan performed upon arrival in the ED, showing hypodensity in the left caudate nucleus; (**B**–**D**) MRI scan (DWI, ADC, FLAIR) performed the day after the patient’s admission in the ED, confirming a subacute ischemia in the left caudate nucleus; the Angio-RM TOF study documented regular flow signal from the major intracranial arterial vessels in the absence of significant dilations/stenosis (these sequences are not shown here); (**E**) CT scan performed during neurological clinical worsening, with a significant decline in consciousness, showing marked dilation of the ventricular system both supra and infratentorial, along with hypodensity of the subependymal white matter, indicating acute communicating hydrocephalus with transependymal CSF reabsorption; (**F**) CT scan performed after the insertion of the EDV. This intervention led to a rapid improvement in the patient’s clinical status and a modest reduction in the size of the lateral ventricles.

Upon presentation to the emergency room, vital signs, including blood pressure, heart rate, and oxygen saturation, were within normal limits. The patient exhibited fever of 39 °C, and neurological examinations revealed a persistent headache, mild ideomotor slowing, abulia, and multidirectional oscillations during gait and the Romberg test. The rest of her clinical examination was unremarkable, with no observable focal neurological deficits, although a potential language barrier may have constrained the neurological assessment. A computed tomography (CT) of the brain revealed an ischemic lesion on the head of the left caudate nucleus (Figure 1A). Subsequently, Magnetic Resonance Imaging (MRI) confirmed a restricted signal in DWI/ADC corresponding to the lesion site, with hyperintensity in FLAIR sequences indicative of a subacute ischemic lesion (Figure 1B,D). Additionally, there was no alteration in the blood–brain barrier after contrast medium administration, and the Time-of-Flight Magnetic Resonance Angiography study documented regular flow signals from the major intracranial arterial vessels in the absence of significant dilations/stenosis (Figure 1B–D). Accordingly, aspirin was administered for the secondary prevention of ischemic stroke. Comprehensive cardiovascular investigations, including echocardiography and Doppler ultrasonography of the supra-aortic trunks, revealed no abnormalities.

The patient continued to experience persistent low-grade fever, night sweats, dry skin, mucosal dryness, and tachycardia, with a heart rate of 115 beats per minute in sinus rhythm. Laboratory tests revealed hyponatremia and lymphocytopenia. Given the suspicion of a viral etiology as the underlying cause of the fever and associated lymphopenia, a comprehensive serological panel was performed. The tests screened for herpes simplex virus type 1 and type 2, Epstein–Barr virus, cytomegalovirus, rubella virus, measles virus, and both human immunodeficiency virus type 1 and type 2. All results were negative. A lymphocyte subset analysis revealed a slight reduction in CD3+ T cells and CD3+ CD4+ T cells. Further investigations, including urine cultures and a chest X-ray, showed no evidence of infection. The urinary antigen test for Legionella was negative. Additionally, nasopharyngeal swabs tested negative for severe acute respiratory syndrome coronavirus, influenza A and B, and respiratory syncytial virus.

**Figure 2:**
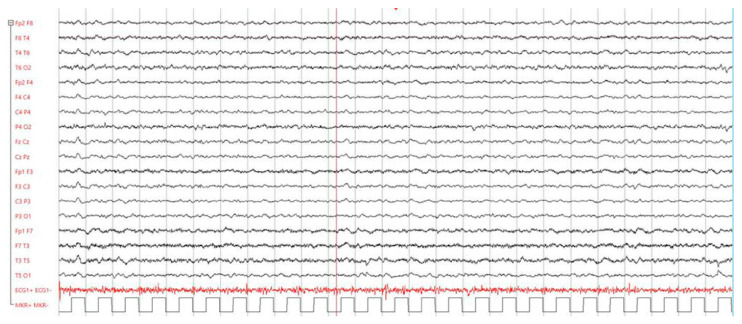
EEG shows slow waves in the anterior territories of both hemispheres.

Due to persistent fever, apathy, ideomotor slowing, and clinical features inconsistent with the location of the ischemic lesion, encephalitis was suspected. An electroencephalogram (EEG) revealed frequent localized slow waves in the anterior regions of both hemispheres, supporting the clinical suspicion of meningoencephalitis (Figure 2). A lumbar puncture was subsequently performed, showing clear cerebrospinal fluid (CSF) with a white blood cell count of 744 cells/mm^3^, predominantly lymphocytic (91%), along with glucose at 2.3 mmol/L, protein at 1659 mg/dL, and lactate at 7.2 mmol/L.

CSF samples were sent for bacterial culture, microscopy, and film array testing, along with Polymerase Chain Reaction (PCR) for Mycobacterium tuberculosis, given the patient’s geographic background. The PCR test confirmed tuberculosis, leading to a diagnosis of tubercular meningitis. Treatment was promptly initiated with rifampicin, isoniazid, ethambutol, pyrazinamide, and linezolid, alongside dexamethasone at 0.4 mg/kg/day [2,3]. Despite the initiation of antitubercular therapy, unexpected and rapid neurological deterioration was observed. A new CT scan of the brain was performed, detecting the presence of acute hydrocephalus (Figure 1F). Hence, an external ventricular drain (EDV) was placed, and follow-up CT scan showed a reduction in ventricular volume and size (Figure 1E).

The day after initiating treatment, the patient’s condition rapidly deteriorated, with a significant decline in consciousness, becoming responsive only to painful stimuli. An emergency brain CT scan revealed significant ventricular dilation in both supratentorial and infratentorial regions, with subependymal white matter hypodensity, indicating acute communicating hydrocephalus with trans-ependymal CSF absorption. An external ventricular drain was urgently placed, resulting in rapid clinical improvement and a modest reduction in lateral ventricular size on subsequent CT imaging (Figure 1E).

To address this critical condition, a neurosurgical procedure was performed to insert an external ventricular drain. This intervention led to a rapid improvement in the patient’s clinical status and a modest reduction in the size of the lateral ventricles on a follow-up CT scan (Figure 1F).

**Figure 3:**
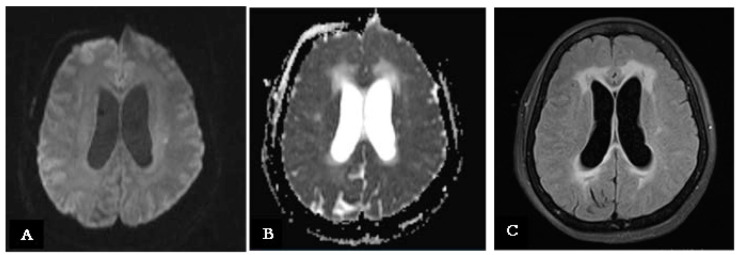
(**A**) A new MRI scan (performed during the stay in the infective disease ward) showing an hyperintensity signal on the left corona radiate on a DWI sequence, indicating a new ischemic lesion. (**B**) Same MRI brain scan on an ADC sequence showing a hypointense signal on the left corona radiate, indicating the same ischemic lesion on left corona radiate. (**C**) Same MRI brain scan on a FLAIR sequence showing a hyperintense signal on the left corona radiate, indicating the subacute evolution of the ischemic lesion.

Several days later, a brain MRI with contrast revealed intense enhancement and thickening of the leptomeninges at the Sylvian fissure, skull base, and truncal and cervical regions. Nevertheless, additional hyperintense areas in diffusion-weighted sequences at the left corona radiata, corresponding to hyperintensity in FLAIR sequences, were found, suggesting a new small subacute ischemic lesion (Figure 3A–C).

### 2.3. Follow-Up and Outcomes

Upon discharge from the Infectious Diseases Department, the patient had difficulty walking due to prolonged bed rest and muscle atrophy, along with mild psychomotor slowing, hand weakness, and dysmetria. A CT scan confirmed proper placement of the ventriculoperitoneal shunt and reduced ventricular size. Three months later, following rehabilitation, the patient regained full walking independence with mild psychomotor slowing and hand weakness persisting. An MMSE score of 25/30 was recorded, likely influenced by language barriers. At the six-month follow-up, walking improved further, and the psychomotor slowing was resolved, with a slight improvement in hand weakness. MRI showed persistent pachymeningeal thickening and ischemic sequelae, with normal ventricular and cerebrospinal fluid spaces. At one year, a neuropsychological assessment showed normal memory, attention, and executive functions, though some challenges in recognizing emotions via facial expressions were noted, possibly due to cultural factors. The patient scored 27/30 on the MMSE and maintained full independence in daily activities.

### 2.4. Patient Perspective

All phases of the diagnostic, therapeutic, and care processes were communicated and shared with the patient and her family whenever possible, given the rapid progression of the disease and its complications.

## 3. Discussion

Approximately 20% of TBM patients experience ischemic neurological deficits, with imaging studies indicating cerebral infarctions in up to 57% of cases. Seventy-five percent of the infarctions were in the “tuberculosis area” encompassing the caudate nucleus, ventral thalamus, and forelimb of the internal capsule [1]. These regions were primarily supplied by the medial columnar artery and thalamic perforating arteries. Meanwhile, 11% of the infarcts occurred in the lateral basal ganglia and the “ischemic region” of the hind limb of the internal capsule, with blood supply from the lateral columnar artery and thalamic artery [9]. According to the literature, autopsy findings indicated that most macro-infarcts in the middle cerebral artery territory were associated with proliferative lesions. Conversely, in regions with a very low artery density, small vascular necrotic lesions with minor infarcts were observed [10].

The exact pathogenesis of stroke in TBM remains uncertain. Some researchers suggest that cerebral infarction in tubercular meningoencephalitis may be caused by vasculitis or intimal hyperplasia, with intra-arterial thrombosis also potentially contributing to the occurrence of stroke [11]. Inflammatory responses triggered by secretion can lead to arteritis and vasospasm in arteries. These conditions may result in intimal hyperplasia, ultimately contributing to the risk of stroke.

Moreover, other hypotheses suggest the development of vasospasm in early stages while later stages may involve localized proliferative intimal reactions that contribute to the occurrence of stroke [11]. The role of cytokines like tumor necrosis factor (TNFα), vascular endothelial growth factor (VEGF), and matrix metalloproteinases (MMPs) in damaging the blood–brain barrier, attracting leucocytes and the release of vasoactive autocoids, potentially, could suggest an arteritis mechanism causing ischemic stroke [12,13]. Cytokines are thought to play a crucial role not only in major complications like stroke and hydrocephalus but also in paradoxical reactions (PRs) [14]. These reactions involve the worsening or emergence of new lesions following the start of antitubercular therapy, even when initial clinical improvements are observed [15,16,17,18]. Some researchers have described what they call the “immunologic paradox”, where an increase in MTB-specific Th1-cell activity is noted in the cerebrospinal fluid (CSF) or peripheral blood after two to four weeks of treatment despite ongoing clinical improvements. This phenomenon is believed to stem from the heightened stimulation of both humoral and cell-mediated immune responses, triggered by antigens released from lysed bacteria [19]. The recurrent nature of these paradoxical effects supports the theory that the immune response is driven by the local release of bacterial antigens. Paradoxical reactions can be categorized based on their timing: they are considered “definite” when complications arise after the fourth week of treatment, while those occurring between the second and fourth weeks are classified as “probable” PRs. Neurological complications during antitubercular therapy are relatively common, as highlighted by several studies. However, it remains uncertain whether our case fits this classification based on temporal and radiological criteria. Specifically, the contrast enhancement of the leptomeninges, the emergence of new ischemic lesions, and hydrocephalus—complications observed in our case—are also frequently seen in tubercular meningoencephalitis, complicating the differentiation from paradoxical reactions [20].

Furthermore, it has been suggested that hypovolemia caused by cerebral salt wasting (CSW) may contribute to the development of stroke in patients with tubercular meningitis (TBM). Ischemic stroke occurs in approximately 39.5% of TBM cases, with CSW present in half of these patients. In such cases, strokes are more frequently localized to ischemic zones, tend to occur in older individuals with traditional cardiovascular risk factors, and often arise during the polyuric phase of CSW [21].

## 4. Conclusions

This report highlights the diagnostic and therapeutic challenges encountered in managing patients with tubercular meningitis complicated by concomitant ischemic stroke. By examining the complexities of this clinical scenario—including a comparative analysis with similar cases from the literature [22,23,24,25,26,27,28] (See Table S1) and a detailed long-term follow-up—we emphasize the importance of early recognition, thorough evaluation, and a multidisciplinary approach to optimize patient outcomes.

Treatment should focus on antimicrobial therapy targeting Mycobacterium tuberculosis and corticosteroids to reduce inflammation and improve survival. Additionally, it is essential not to overlook the hypercoagulable state associated with tubercular meningitis. Aspirin use in patients with tuberculous meningitis (TBM) for stroke prevention may increase the risk of gastrointestinal and cerebral bleeding; however, the overall benefits appear to outweigh these potential risks [29]. Bleeding rates in aspirin-treated patients are not significantly higher than in those receiving placebo. Furthermore, aspirin has been shown to reduce short-term mortality, despite not having a significant effect on stroke prevention [30]. These findings support the use of aspirin as a beneficial adjunctive therapy in TBM, provided there are no clear signs of major bleeding risk. Furthermore, studies have indicated that corticosteroids may help reduce mortality in stroke patients [31]. 

Finally, careful monitoring for the emergence of new neurological symptoms or brain lesions is essential both during hospitalization and throughout follow-up. The timing of these manifestations can provide valuable insights into whether they represent paradoxical reactions or the progression of previously latent or radiologically occult lesions. Ongoing clinical and imaging surveillance is, therefore, critical for the accurate interpretation and timely management of such complications.

## Figures and Tables

**Table 1:** Chronological summary of clinical manifestations, diagnostic workup, and therapeutic interventions. This table outlines the patient’s clinical course from admission through diagnosis and treatment. Progressive neurological deterioration prompted extensive diagnostic evaluation, leading to the diagnosis of tubercular meningitis complicated by ischemic stroke and hydrocephalus. Key milestones include CSF analysis, imaging findings, and the initiation of antitubercular therapy and neurosurgical management.

Date	Clinical Findings	Diagnostic Exams and Results
Day 0	Generalized weakness, fever (39 °C), persistent headache.	CT scan: Ischemic lesion in the head of the left caudate nucleus.
Day 1–3	Worsening ideomotor slowing, apathy, abulia, night sweats, serotine fever (38.5 °C), tachycardia (HR 115 bpm), fatigue.	Tests: Negative for HSV 1 and 2, EBV, CMV, rubella, measles, HIV, Legionella, SARS-CoV-2, flu A/B, RSV. Urine culture: Negative. Chest X-ray: No abnormalities. Lymphocyte subset: Slight reduction in CD3+ and CD3+ CD4+ T cells. Echocardiography and Doppler ultrasonography of the supra-aortic trunks: Unremarkable.
Day 4	Continued apathy, ideomotor slowing.	EEG: Localized slow waves in anterior regions, suggestive of meningoencephalitis.
Day 5	Neurological symptoms progressively worsened: increased ideomotor slowing, apathy, abulia, and new symptoms such as Romberg oscillations, cautious gait, and vertigo.	Lumbar puncture: Lymphocytic predominance in CSF.
Day 6	Further worsening of neurological symptoms, with continued ideomotor slowing.	TB Diagnosis: Mycobacterium tuberculosis in CSF. Antitubercular therapy started.
Day 7–8	Transferred to Infectious Diseases Department. Progressive decrease in arousal, leading to coma.	CT scan: Acute hydrocephalus detected. Neurosurgery: External ventricular drain inserted urgently.
Day 9	Improvement in consciousness level. Continued ideomotor slowing, apathy, and abulia.	MRI with contrast: Leptomeningeal enhancement and new ischemic lesion at left corona radiata; hydrocephalus with signs of recent transependymal resorption.

## Data Availability

The original contributions presented in this study are included in the article/Supplementary Material. Further inquiries can be directed at the corresponding author.

## Supplementary Materials

The following supporting information can be downloaded at https://www.mdpi.com/article/10.3390/reports8020055/s1, Table S1: Summary of key case reports on tuberculosis-related cerebral infarction.

## Abbreviations

The following abbreviations are used in this manuscript:

CSF	cerebrospinal fluid
CT	computed tomography
EEG	electroencephalogram
MRI	Magnetic Resonance Imaging
PCR	Polymerase Chain Reaction
TB	tubercular
TBM	tuberculous meningitis
EDV	external ventricular drain
VP	ventriculoperitoneal shunt
