# Supplementary material for: Caudate Head Ischemic Stroke with Concurrent Tubercular Meningoencephalitis: A Case Report
Source: Reports (MDPI). 2025 Apr 23;8(2):55. doi: 10.3390/reports8020055 (PMC12197144; doi:10.3390/reports8020055)
Supplement: Supplementary file 1 [file reports-08-00055-s001.zip › reports-3515897-supplementary.pdf]

Supplementary Table S1

Study	Patients feature	Stroke localization	Follow up / outcome
Katwal et al., (2020)	25-year-old M. No significant prior medical history	Basal ganglia, anterior and posterior limb of left internal capsule	6 months / significant recovery with mild residual weakness.
Bhusal et al., (2020)	32-year-old male. Untreated pulmonary tuberculosis	Right internal capsule, left lentiform nucleus, genu of corpus callosum.	3 months / partial recovery, mild weakness and speech deficits remained.
Razmeh et al. (2017)	27-year-old male. No significant prior medical history	Pons, midbrain, temporal and internal capsule	6 months /partial recovery, persistence of mild motor deficits remained
Pasticci et al., (2013)	45-year-old male. HIV and tuberculous meningitis	Left temporal-parietal lobe	4 months / partial recovery with mild residual deficits.
Peng et al., (2021)	50-year-old male. No significant prior medical history	Left frontal and insular lobes	Not specified; patient discharged with mild facial paralysis.
Mengst et al. (2023)	35-year-old male. Tuberculosis epididymo-orchitis	Anterior left thalamus and temporal-parietal lobe	Not specified
Aasfara et al., (2017)	15- year- old male. No significant prior medical history	Bilateral thalamus	3 months / partial recovery, cognitive impairment, focal dystonia

Summary of key case reports on tuberculosis-related cerebral infarction. Stroke localization varies significantly among cases, and follow-up data are limited, with a maximum duration of six months reported. Notably, our case stands out due to the extended clinical and radiological follow-up, complemented by a complete neuropsychological evaluation.
